# Automatic DNA Diagnosis for 1D Gel Electrophoresis Images using Bio-image Processing Technique

**DOI:** 10.1186/1471-2164-16-S12-S15

**Published:** 2015-12-09

**Authors:** Apichart Intarapanich, Saowaluck Kaewkamnerd, Philip J Shaw, Kittipat Ukosakit, Somvong Tragoonrung, Sissades Tongsima

**Affiliations:** 1 National Electronics and Computer Technology Center (NECTEC), National Science and Technology Development Agency (NSTDA), Pathum Thani, Thailand; 2 National Center for Genetic Engineering and Biotechnology (BIOTEC), National Science and Technology Development Agency (NSTDA), Pathum Thani, Thailand; 3 Faculty of Science and Technology, Thammasat University, Pathum Thani, Thailand

**Keywords:** GELect, DNA Gel electrophoresis, Genotyping, Sugarcane, Image processing, Automation

## Abstract

**Background:**

DNA gel electrophoresis is a molecular biology technique for separating different sizes of DNA fragments. Applications of DNA gel electrophoresis include DNA fingerprinting (genetic diagnosis), size estimation of DNA, and DNA separation for Southern blotting. Accurate interpretation of DNA banding patterns from electrophoretic images can be laborious and error prone when a large number of bands are interrogated manually. Although many bio-imaging techniques have been proposed, none of them can fully automate the typing of DNA owing to the complexities of migration patterns typically obtained.

**Results:**

We developed an image-processing tool that automatically calls genotypes from DNA gel electrophoresis images. The image processing workflow comprises three main steps: 1) lane segmentation, 2) extraction of DNA bands and 3) band genotyping classification. The tool was originally intended to facilitate large-scale genotyping analysis of sugarcane cultivars. We tested the proposed tool on 10 gel images (433 cultivars) obtained from polyacrylamide gel electrophoresis (PAGE) of PCR amplicons for detecting intron length polymorphisms (ILP) on one locus of the sugarcanes. These gel images demonstrated many challenges in automated lane/band segmentation in image processing including lane distortion, band deformity, high degree of noise in the background, and bands that are very close together (doublets). Using the proposed bio-imaging workflow, lanes and DNA bands contained within are properly segmented, even for adjacent bands with aberrant migration that cannot be separated by conventional techniques. The software, called GELect, automatically performs genotype calling on each lane by comparing with an all-banding reference, which was created by clustering the existing bands into the non-redundant set of reference bands. The automated genotype calling results were verified by independent manual typing by molecular biologists.

**Conclusions:**

This work presents an automated genotyping tool from DNA gel electrophoresis images, called GELect, which was written in Java and made available through the imageJ framework. With a novel automated image processing workflow, the tool can accurately segment lanes from a gel matrix, intelligently extract distorted and even doublet bands that are difficult to identify by existing image processing tools. Consequently, genotyping from DNA gel electrophoresis can be performed automatically allowing users to efficiently conduct large scale DNA fingerprinting via DNA gel electrophoresis. The software is freely available from http://www.biotec.or.th/gi/tools/gelect.

## Background

DNA gel electrophoresis (GE) technology is a method to separate DNA molecules by their size. This technology has a wide number of applications, including size estimation of DNA molecules [1], analysis of PCR amplicons or genotyping [2], and separation of genomic DNA before Southern analysis [3]. To perform genetic diagnosis, target DNA sequences are amplified by polymerase chain reaction (PCR). The resulting PCR products (amplicons) are loaded into wells located on top of the gel matrix that indicate lanes for DNA molecules to migrate through the gel medium. At the end of electrophoresis, different sizes of DNA molecules appear as bands in each lane. These bands can be visualized by DNA stains such as ethidium bromide (agarose gel) or silver nitrate (polyacrylamide gel). A densitometer is commonly used to capture the band images from the gel slab. Manual interpretation of banding patterns can be very laborious and inaccurate. Performing large-scale DNA fingerprinting or genotyping thus requires an automated workflow for analysis.

Many imaging processing techniques have been proposed to address the two main steps in GE analysis, namely lane and band detection. The accuracy of these steps is often compromised by technical variation inherent to GE [4]. This variation includes distortion, i.e. lane or band curvature, which affects automatic lane segmentation, and sub-optimal gel image exposure that affects band detection performance. Caridade et al., [5] presented a technique to extract DNA bands by converting an input image to gray scale and using the column histogram method to detect lanes. To detect DNA bands, they proposed a heuristic to match a given band to a reference band. The band quantification accuracy of this technique is very variable among GE images. Bajla et al. [6] proposed a technique to deal with image distortion by letting users to adjust a Gaussian deconvolution parameter so that band positions can be easily detected. Kaabouch et al. [7] attempted to improve the band detection process by enhancing the quality of a gel image first using their proposed automatic thresholding technique. Lee et al. [8] presented another automated gel electrophoresis analysis system that uses an enhanced fuzzy *c*-means algorithm and Gaussian function for lane segmentation. In their workflow, the bands were identified by tracing the segmented lanes while enhancing the detection accuracy through an elimination of repetitive band procedure. The Dynamic Time Warping (DTW) method was introduced in [9] to increase band detection sensitivity by cross-adjusting positions of the same bands from different lanes. A recent report by Tseng and Lee [10] claimed that none of the previously presented techniques can fully automate the band detection process. They offered new heuristics that can adjust for geometric distortion of lanes (slanted lanes) and increase the sensitivity of band identification by taking first derivative of the band gray-level. Doublet bands (two bands that are very close together in a lane) can be extracted with high accuracy by this method.

Although most research efforts claimed to have an automated band extraction system, none of them offer practical software that can be used to carry out the underlying task. Tseng and Lee [10] established the theoretical platform of image processing techniques that could be implemented as an automated tool. Several commercial software tools such as GelQuant, QuantiScan, Gel-Pro Analyzer and GelCompar [11-14] offer a partial image processing solution with limited features. The review article by Heras et. al. [15] surveys DNA fingerprinting tools, including Gel Plugin ImageJ [16], GelAnalyzer [17], GelClust [18], GelQuant.NET [19], Image [20], Laneruler [21] and PyElph [22]. Several of these free tools, however, either have limited function (GelQuant.NET has no lane detection module) or can no longer be used owing to outdated dependent software (Image software by Sanger and Laneruler). Moreover, the lane analysis available in Gel Plugin ImageJ does not have automatic lane detection. The most recently published tool GelJ [23] provides a comprehensive tool incorporating many features of DNA fingerprinting available in other tools.

The performance of these image processing tools depend majorly on the ability to detect lanes correctly. Most tools assume that lanes are parallel lines. However, uneven heating or buffer degradation during electrophoresis can often create migration artifacts that lead to lanes that are not straight. The most recent algorithm described in [10] addresses this issue by applying geometric distortion in which a box is created automatically with slanted sides over the lane. This method can correct for minor lane aberrations. However, we found that this method often fails when lanes are highly curved. We propose a novel image processing tool for gel electrophoresis, called GELect that can automatically perform the analysis of large-scale DNA fingerprinting. In particular, a novel lane segmentation algorithm is incorporated for accurately assigning bands into lanes, even when the lanes are highly curved. Moreover, GELect also offers a genotyping feature that collectively groups the same banding patterns together. We used images obtained from DNA fingerprinting of sugarcane DNA samples to test GELect. To demonstrate the performance over existing tools, we compare GELect with free software, namely PyElph, GelJ, GelClust and GenAnalyzer, in terms of the ability to detect and correct for curved lanes. GELect was implemented in Java and converted into imageJ library so that the tool can be easily utilized as well as further improved by other developers.

## Results and Discussion

We tested the performance of the proposed system in two aspects, lane segmentation and band extraction performance. Ten PAGE images with 433 samples (lanes) were tested on both aspects. We examined how well the proposed system is able to separate distorted lanes. After performing lane separation, each lane was further analyzed to detect DNA bands.

The proposed algorithm for segmenting curved lanes was able to completely separate lanes that cannot be formed by two parallel lines (Table 1 and Additional File 1). In these cases other tools are not able to correctly assign lanes using their automatic lane assignment feature (Additional File 2). It should be noted that the performance of these tools can be optimized using the manual adjustment features incorporated in them. To allow a fair comparison, we employed only automated features under their default settings. Although GELect was shown to be superior to all other tools for automatic curved lane detection, the performance of GELect for detecting lanes was rather poor in some images where the lanes had very few bands. In this case, the automatic lane de-tection works poorly because there are insufficient bands for the program to join segments together correctly in the same lane. In this case, it may be more useful to employ a straight lane detection for delimiting lanes, which would work even when the lane is devoid of bands. Hence, we offer an option for users to select if they want to use the curved lane or straight lane detection algorithm in GELect to accommodate this shortcoming. The curve lane assignment could be further improved by incorporation of curvilinear fitting as used in manually drawing feature in GelJ.

**Table 1:** Comparison of different gel analysis tools.

	Lane detecttion	Curved Lane detection	Band smiling effect correction	Band detection	Dendrogram
PyElph	Yes (Auto)	No	Yes (Manual)	Yes (Auto)	Yes (Auto)

GelJ	Yes (Auto)	Yes (Manual)*	Yes (Auto)	Yes (Auto)	Yes (Auto)

GelClust	Yes (Auto)	No	Yes (Auto)	Yes (Auto)	Yes (Auto)

GelAnalyzer	Yes (Auto)	No	Yes (Manual)	Yes (Auto)	No

GELect	Yes (Auto)	Yes (Auto)	Yes (Auto)	Yes (Auto)	No

Four free gel analysis software were compared in terms of their functions, namely lane detection, ability to detect curved lanes, ability to correct smile/frown effect, band detection and construction of dendrogram.

^* ^GelJ can perform curved lane detection using curvilinear models, e.g, cubic spline to assist user to manually draw lane boundaries.

To demonstrate the need of curved lane detection, we also compared GELect with PyElph, GelJ, GelClust, and GelAnalyzer in terms of their ability to segment curved lanes (Table 1 and Additional File 1). GelJ allows users to manually draw polygons to select the lanes. However, we did not test this function as we were only interested in comparing the automatic feature of each al gorithm. Of these tools, only GELect can automatically detect curved lanes. Other tools use the as sumption that lanes can only be constructed by two parallel lines.

## Conclusion

The GELect tool is a convenient program for DNA diagnosis from 1D gel electrophoresis image. The tool can efficiently segment lanes from gel electrophoresis image with curved lanes as well as poor image exposure. GELect can construct a band model by performing band registration against a reference band. Therefore, the genotyping from DNA gel electrophoresis can be done through the band classification technique.

## Materials and methods

### Genotyping of sugarcane cultivars

We obtained 433 sugarcane cultivars from Mitr Phol research [24] with different phenotypes including sweetness, measured in cane content sugar (CCS), capacity to produce biomass, measured by weight and other. The genotyping locus was chosen from the known sugarcane ESTs that were predicted to have an important sucrose metabolism (sbi00500) function by performing BLASTX of the EST sequences against the annotated sorghum genome from the Phytosome database [25]. Both reverse and forward primers were designed using Primer3 to amplify intron amplicons. Polymerase chain reaction (PCR) was conducted in 25 µl final volumes comprising: genomic DNA 5 ng; 1X PCR buffer; 25 mM MgCl2; 0.2 mM each dNTP, 0.32 µM each primer and 0.04 U *taq *DNA polymerase. The thermocycling conditions for PCR were: 35 cycles of 94C for 3 min, 72 C for 5 min. Agarose gel electrophoresis (AGE) with 2% gel (TBE buffer) was used to demonstrate if intron length polymorphisms (ILPs) present. Genotyping of 433 cultivars was done using polyacrylamide gel electrophoresis (PAGE) with 5% gel (TBE buffer) staining fragments with silver nitrate. PCR products of 433 samples were separated in 10 slab gels. Densitometry was performed on the stained gels producing 8-bit gray scale images (see Additional File 3 for the PAGE images). The in formation about these images is shown in Table 2.

**Table 2:** The lane segmentation results.

Test image	Dimension in pixels	Number of lanes	Detection results	Accuracy in %
1a	1884 × 524	72	39	54.16

2a	1955 × 524	60	37	61.67

3a	1871 × 524	72	19	26.83

4a	1911 × 546	60	45	75.00

5a	1810 × 718	56	46	82.14

6b	1810 × 718	34	34	100

7b	473 × 288	32	31	96.87

8b	234 × 500	15	14	93.33

9b	276 × 574	15	15	100

10b	276 × 399	17	17	100

Ten electrophoretic gel images are used to test the performance of the proposed lane detection. aImages 1-5 contain lanes that can be formed by using two parallel lines. bImages 6-10 contain curved lanes.

### Overview of image processing workflow

**Figure 1:**
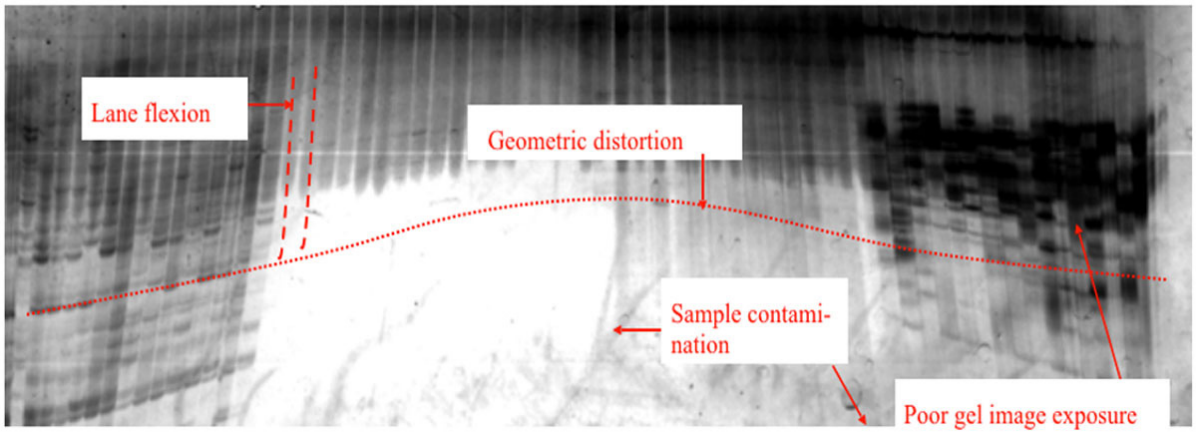
**Distortion in gel electrophoresis**. A sample of gel electrophoresis image reveals common challenges for image processing, including geometric distortion, lane flexion, low contrast region and artifacts due to sample contamination.

**Figure 2:**
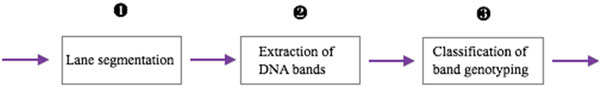
**The Overview of GELect**. GELect workflow comprises three main procedures: 1) lane segmentation, 2) DNA band extraction and 3) band genotyping.

In GE images, the image geometry is often distorted as shown in Figure 1. In particular, some or all lanes on the image are not uniformly straight, e.g., outward curving lanes, inward curving lanes and slanted lanes. This lane distortion is a common problem that may stem from various factors including gel environment and buffer type. Thus, a flexible lane segmentation algorithm that can precisely identify lanes and extract as much band information as possible is preferable. All previously reported lane segmentation techniques [5,6] make the erroneous assumption that two parallel lines can be formed to segment a lane. For band identification, both distortion and ambiguity patterns exist such as smiling/frowning bands, low contrast bands, noisy background, and doublet bands etc. Most reported heuristics recommend performing image enhancement and background removal in order to highlight these bands [7-9]. However, doublet bands, i.e. two bands of very similar mobility, cannot be separated by previous band detection approaches. Both lane segmentation and band extraction routines (Figure 2) are explained as follows:

#### Lane segmentation

**Figure 3:**
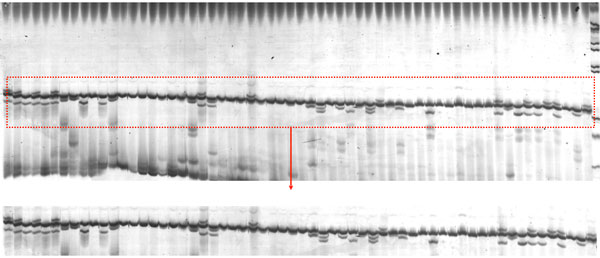
**Cropping gel image**. The region of interest must be cropped by users to be analyzed by GELect.

Gel electrophoresis images were taken using a GS-800 calibrated densitometer (Bio-Rad). The optimal resolution of DNA bands is obtained in the middle of lanes where migration most closely follows the linear relationship to log molecular weight. We assume that the users have already optimized their electrophoresis protocol so that the bands of interest are resolved in this region and that this part of the image can be isolated for analysis by cropping (Figure 3). Cropping should also be performed to remove regions of gel that shows extreme artifacts that could interfere with band detection, e.g., severely distorted lanes with no discernible bands. Let us assume that a generic 1D gel image has the layout as shown in Additional File 4. Each box represents a pixel in this image.

Consider the situation where lanes are not straight, such that a vertical line drawn through bands from one lane in the upper portion of the gel image do not pass through bands migrating in the same lane in a lower portion of the image. To address this problem, the input image is first separated into *N *strips with sides *H_i _*and equal width *W*. This is the major novelty in our approach that allows us to detect lanes that may be distorted, such that the register of lanes can shift laterally from one portion of the gel image to another. The height of the strips *H_i _*is determined according to the local contrast in the gel image. For example, regions of the gel with intensely staining bands will have high local contrast, whereas other regions with few bands will have low contrast. The height *H_i _*of a particular strip is determined using three steps: calculation of cumulative pixel intensity (*c*), curve smoothing and lane width estimation. The cumulative pixel intensity is calculated horizontally across the strip one pixel at a time. The cumulative intensity of the *n*^th ^pixel column, *c*_i_(*n*) can be calculated by taking the summation of pixel intensity values (*p*) along *H*_i _as follows:

(1) $$c_{i}\left(n\right)=\overset{H_{i}}{\underset{m=1}{\sum }}p_{mn}$$

Note that *H*_i _segment height is dynamic according to the banding pattern and can be easily computed by using cumulative row intensity similar to Equation 1 as follows:

(2) $$r\left(m\right)=\overset{W}{\underset{n=1}{\sum }}p_{mn}$$

**Figure 4:**
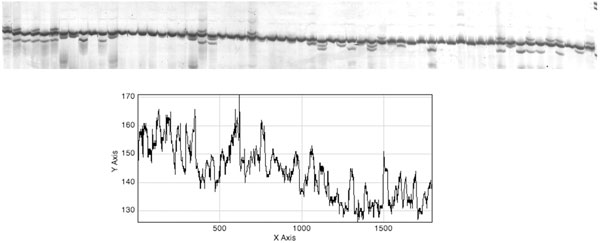
**Cumulative pixel intensity histogram before smoothing**. Each point is obtained by summing the intensity values of all pixels along vertical line the above gel image.

The distance separating two regions of high contrast in the *r*(*m*) plot defines the *H*_i _side. For each *H*_i_, the cumulative pixel calculation is performed at consecutive pixels along the width of the gel. The values of *c*_i_(*n*) and *r*(*m*) can be plotted (Figure 4). It is difficult though to identify the lane edge from this irregular distribution. Hence, curve smoothing must be performed in the second step. The following equation does a simple smoothing process by taking the average of every three consecutive pixel intensities as shown below:

(3) $${\bar{c}}_{i}\left(n\right)=\frac{1}{3}\overset{3}{\underset{k=1}{\sum }}c_{i}\left(n+k\right)$$

**Figure 5:**
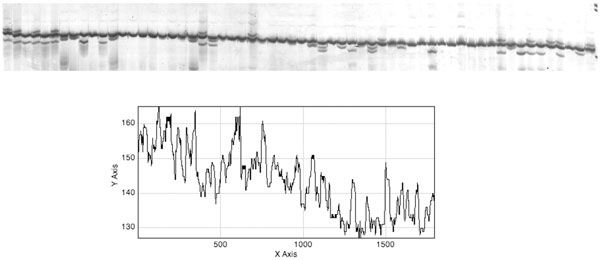
**Smoothed cumulative pixel intensity histogram**. After applying the smooth filter (average signal intensity of three adjacent cumulative pixel intensity values), the peaks and valleys that demarcate the underlying lanes become more apparent.

**Figure 6:**
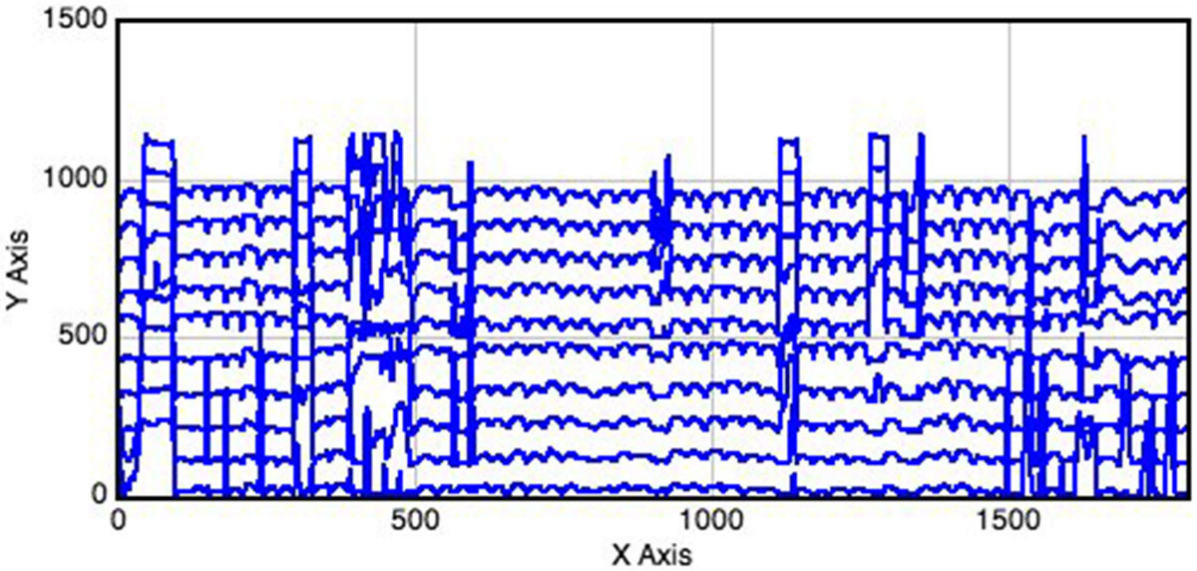
**A stack of cumulative pixel intensity histogram**. Histograms from different *H_i _*are plotted together on the same axes. Valleys from different strips (histogram stack) must be joined using the shortest path heuristic (see Figure S3 on how lane boundaries are drawn).

After smoothing, the peaks and valleys can be observed more clearly (Figure 5). It is observable that the high cumulative intensity peaks represent the regions where lane boundaries are present. Hence, the steep peaks between valleys help locate the lane boundaries. The smoothed histograms of cumulative pixel intensity constructed for all *H*_i _strips are then plotted on the same axes (Figure 6). To register the lanes among strips, we need to find a way to stitch all the lane segments from *H*_1 _to *H*_N _for the next band extraction procedure. This is performed by finding the shortest path from a valley in one strip to the next (Figure 6). Examples of lane detection in test images are shown in Additional File 5. Once lanes have been detected, the algorithm proceeds to the next step of locating bands.

#### Extraction of DNA bands

**Figure 7:**
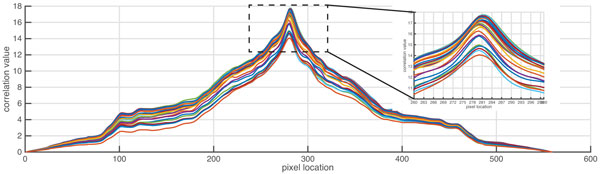
**The cross correlation between column indexes for each lane**. To correct for band distortion within a lane, e.g., smiling, the GELect algorithm calculates correlation of pixel intensities across the lane. The pixel offset for each column of one pixel width can be detected and corrected for. In the example plot shown, a lane of 15 pixels width contains bands with a frowning pattern. It can be seen that the peak correlation values plotted as pairwise correlations shift along the vertical lane axis (see inset).

Before proceeding to detection of DNA bands, the users must be satisfied that regions of the gel image that could interfere with band detection have been removed by cropping (see above) and lanes have been correctly assigned. The band extraction comprises two steps: intra-lane alignment of bands and band assignment. In most GE images, DNA bands are not straight, i.e., slanted, smiling/frowning bands. To make a straight band, the entire pixel column of defined height must be shifted (either by moving pixels up or down) in order to straighten the distorted bands. The cross-correlation product *R*(*k*) is used to measure the similarity of a pair of pixels from two columns. *R*(*k*) is a summation of the inner product between the pixel intensity from the 1^st ^column of a detected reference lane (*p*_(*h*+*k*)1_) and that from the n^th ^column (*p_hn_*), where (*h*+*k*) represents the pixel row (*h*) that is shifted by the *k *offset (Equation 4). A graph of *R*(*k*) values can be plotted when shifting the *k *offset from -*H *to *H *(see Figure 7).

(4) $$R_{1n}\left(k\right)=\left\{\begin{array}{cc} \overset{H-1}{\underset{h=0}{\sum }}p_{\left(h+k\right)1}p_{hn} & k\geq 0\\ R_{1n}\left(k\right) & k<0 \end{array}\right);\;\forall k=\left[-H,\ldots ,H\right]$$

**Figure 8:**
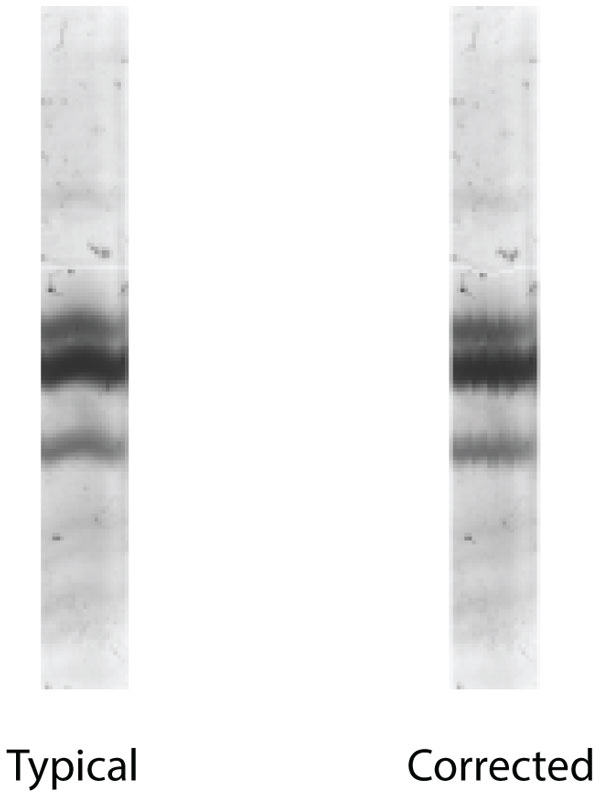
**The distortion correction**. The left image represents the lane of interest that was analyzed as shown in Figure 7. The lane after correction by the algorithm is shown in the right.

If both pixels *p*_*(h+k)*1 _and *p_hn _*belong to the same band, this will result in a higher correlation value (see Figure 7). The cross-correlation adjustment is robust for all bands in the lane, as shown in Figure 8. Similar to the lane detection module, we adopt the cumulative pixel column intensity (band intensity) calculation to reveal band locations. For simplicity in calculation, the vertical lane is rotated 90 degree counter-clockwise. The band intensity on the i^th ^lane (*b_i_*) can be computed by:

**Figure 9:**
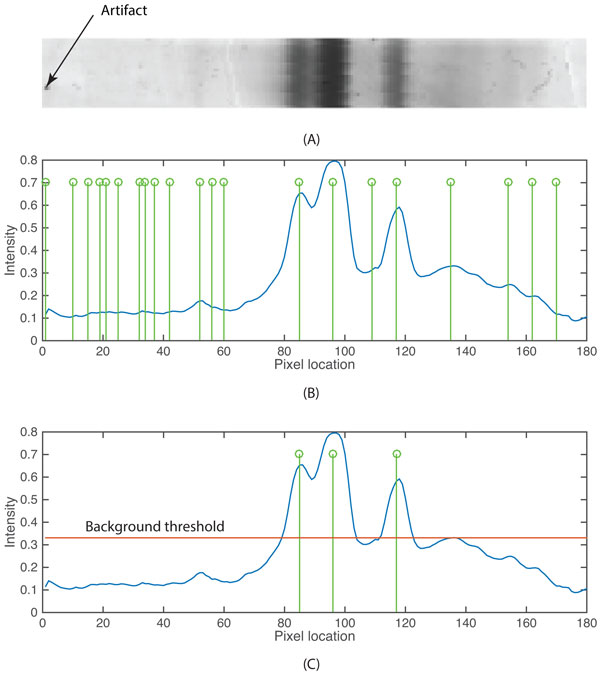
**Band detection within a lane**. (A) The image of a lane with three bands, two of which migrated as a doublet, and some minor artifacts. (B) The blue curve is the summed pixel intensities of a lane and the green stems are possible band locations detected as the first order derivatives. (C) The three green stems are genuine band locations that pass the background threshold (red line).

(5) $$b_{i}\left(n\right)=\overset{W_{i}}{\underset{m=1}{\sum }}p_{mn}^{i}$$

where $p_{mn}^{i}$ is pixel intensity of the i^th ^lane and *W_i _*is the width of the i^th ^lane. Gel artifacts, e.g., dust speckles can be distinguished from genuine bands using peak finding of summed pixel intensities. The first order derivatives are calculated for determining potential peak (band) locations (Equation 6). A threshold of the fifteenth percentile of summed pixel intensities is used to assign genuine bands among the peaks detected.

(6) G(n)=b(n+1)-b(n);n=[1,…,H-1]

#### Automatic band genotyping

A common application of gel analysis includes genotyping in which bands of a certain mobility are associated with common DNA fragments. This process is subject to error both systematic and ran dom. Systematic errors including lane-to-lane variations can be corrected by the algorithm. All lanes must be aligned so that we can register all the bands to have the same relative mobilities among lanes. Similar to the intra-lane alignment where pixel columns are shifted to form a straight band, we could intuitively deploy global inter-lane alignment to first adjust the lane offset using cross correlation calculation as follows:

(7) $$R_{1j}\left(k\right)=\left\{\begin{array}{cc} \overset{H-1}{\underset{n=0}{\sum }}b_{1}\left(n+k\right)b_{j}\left(n\right) & k\geq 0\\ R_{1j}\left(k\right) & k<0 \end{array}\right);\;\forall k=\left[-H,\ldots ,H\right]$$

**Figure 10:**
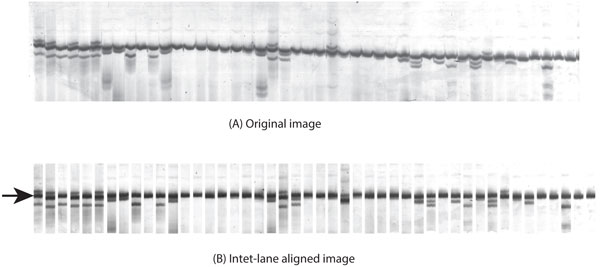
**The inter-lane alignment image**. The upper image (A) is the original image with alignment distortion and the lower image is the aligned image (B). The reference band in each lane used for alignment is indicated by an arrow.

Note that *R*_1*j *_represents cross-correlation between the summed band intensities of the 1^st ^lane (*b*_1_) and that of the j^th ^lane (*b*_j_), where *k *is the shifting offset and *n *is a position on the summed band intensities. A reference band--a band that is always present in all lanes and has very similar mobility in all the lanes is needed so that a *local *cross correlation can be performed relative to the reference band. The reference band must be designed in the electrophoresis protocol. This reference band could be an amplicon that is consistently obtained in all samples, or could represent a "spike-in" DNA species of known sequence. An example of inter-lane alignment using a reference band is shown in Figure 10.

**Figure 11:**
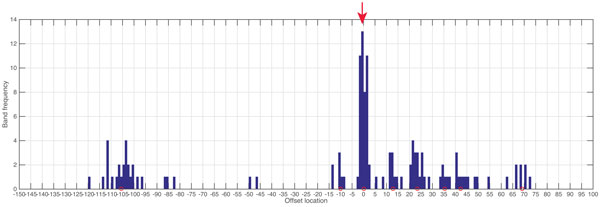
**The band location of all lanes**. The graph shows a histogram of band frequencies across multiple lanes of the same gel. Offset location (*x*axis) is the band mobility relative to the reference after the lane alignment. The reference band is marked by an arrow and it is assigned to have zero offset location. Cluster centroids of bands belonging to common DNA species among lanes identified by DBSCAN are indicated by red circles.

After the lanes have been aligned, the next step is determination of band mobilities relative to the reference band in each lane. As explained above, bands of similar mobilities among lanes often represent the same DNA species, e.g., a genotype. However, the error in electrophoretic mobility makes it difficult to assign bands to DNA species. To assist in this difficult task, we use DB-SCAN, a density-based clustering method [26]. DBSCAN requires two parameters: *ε *and minPts. The first parameter *ε *is the distance threshold used to determine the minimum distance away from the reference for detecting clusters. minPts represents the minimum number of data points (bands) to form a cluster. In our band registration application, *ε *is the range of reference band mobilities among all lanes. The minPts parameter was set to be the integer closest to 10% of the number of lanes. An example of band assignment using DBSCAN across lanes is shown in Figure 11. This step is only needed to be performed once among a group of related gel images/experiments. From the frequency histogram, the mean and variance can be calculated for each band cluster. From these parameters, standard Gaussian classification based on maximum likelihood can be used to assign bands to band clusters. This step corrects for gel-to-gel systematic errors.

### Availability of supporting data

The instruction of the software and the electrophoretic gel images used in this paper are available to download from our website, http://www4a.biotec.or.th/GI/tools/gelect.

## Competing interests

The authors declare that they have no competing interests.

## Authors' contributions

SK, AI, PJS, KU, SVT and SDT conceived the idea of making the automated genotyping tool. KU and SVT carried out the gel electrophoresis experiments and obtained the images used in this work. SK, AI and SDT introduced the idea of object-based approach and refined the algorithm to achieve good performance. SDT, SK, PJS and AI wrote the manuscript. AI and SK created a prototype of this tool in Matlab. The Matlab code was later converted into ImageJ plugin by AI.

## Supplementary Material

Additional file 1 **Figure S1 - Performance of DNA fingerprinting tools for automatic assignment of lanes**. Ten test images were processed and analyzed using the software tools, PyElph, GelJ, GelClust, GelAnalyzer, and GELect, using their default settings. The assigned lanes are shown by the overlaid lines. Click here for file

Additional file 2 **Table T1 - Automated lane identification results**. Test images shown in Additional File 5: Figure S4 were analyzed using the DNA fingerprinting programs under their default settings for automated lane identification. Click here for file

Additional file 3 **Figure S2 - Electrophoresis images used to evaluate lane detection performance**. 10 electrophoresis images are used to test the lane detection feature in GELect. Click here for file

Additional file 4 **Figure S3 - A diagram shows pixel layout in a typical gel electrophoresis image**. Each box represents a pixel in a typical GE image. The image is separated into N strips with sides Hi and equal width W. Click here for file

Additional file 5 **Figure S4 - Lane detection results on the 10 experimental GE images**. A blue line is used to connect two red dots (obtained from histogram peaks). The two red dots that form the shortest path between adjacent strips (Hi and Hi+1) will be connected using a blue line segment. Click here for file

## Declarations

Publication charges of this work were funded by the National Science and Technology Development Agency (NSTDA).

This article has been published as part of *BMC Genomics *Volume 16 Supplement 12, 2015: Joint 26th Genome Informatics Workshop and 14th International Conference on Bioinformatics: Genomics. The full contents of the supplement are available online at http://www.biomedcentral.com/bmcgenomics/supplements/16/S12.
