# Supplementary material for: Automatic DNA Diagnosis for 1D Gel Electrophoresis Images using Bio-image Processing Technique
Source: BMC Genomics. 2015 Dec 9;16(Suppl 12):S15. doi: 10.1186/1471-2164-16-S12-S15 (PMC4682448; doi:10.1186/1471-2164-16-S12-S15)
Supplement: Additional file 1 — Figure S1 - Performance of DNA fingerprinting tools for automatic assignment of lanes. Ten test images were processed and analyzed using the software tools, PyElph, GelJ, GelClust, GelAnalyzer, and GELect, using their default settings. The assigned lanes are shown by the overlaid lines. [file 1471-2164-16-S12-S15-S1.pdf]

Figure S1 Comparison of lane detection feature from different DNA fingerprinting tools

Ten test images were processed and analyzed using the software tools, PyElph, GelJ, GelClust, GelAnalyzer, and GELect, using their default settings. The assigned lanes are shown by the the overlaid lines.

Software name	light band with dark background	dark band with light background
PyElph	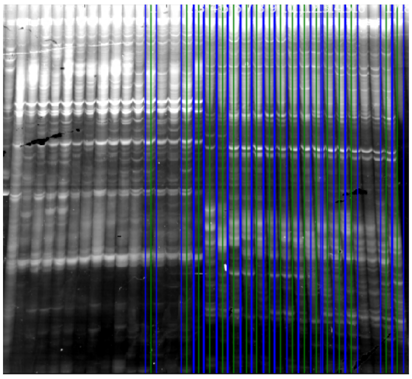	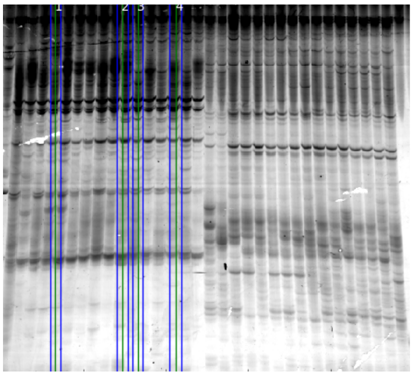
GelJ	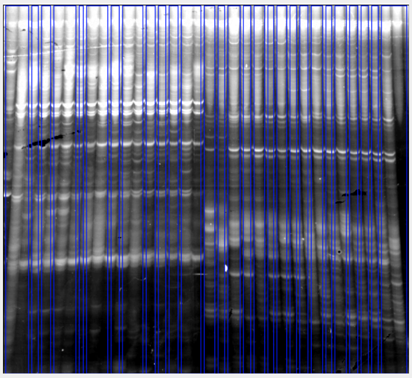	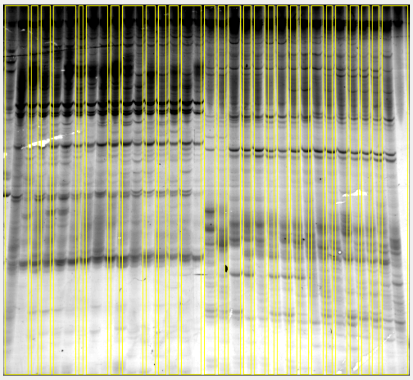
GelClust	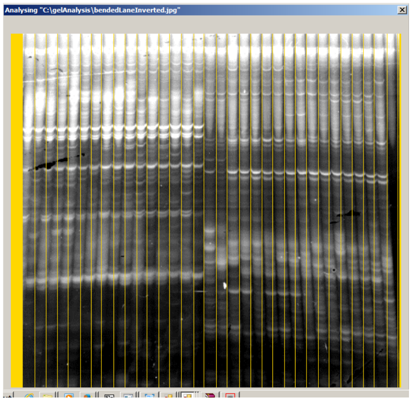	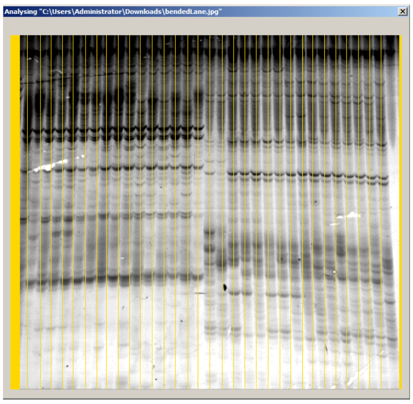
GelAnalyzer	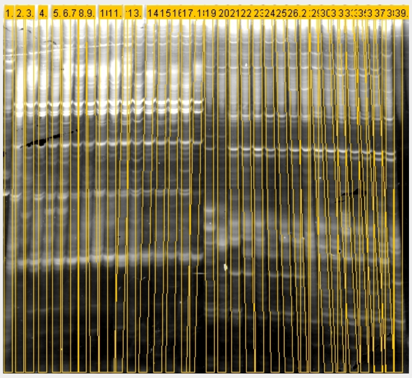	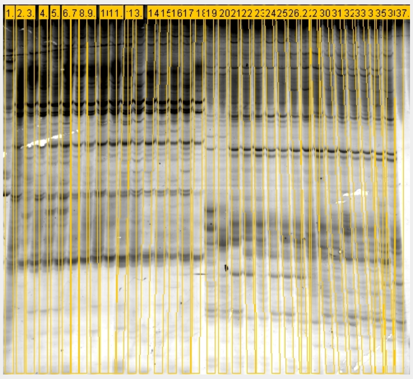

Software name	light band with dark background	dark band with light background
GELect	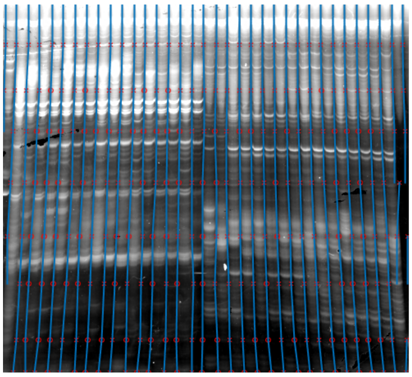	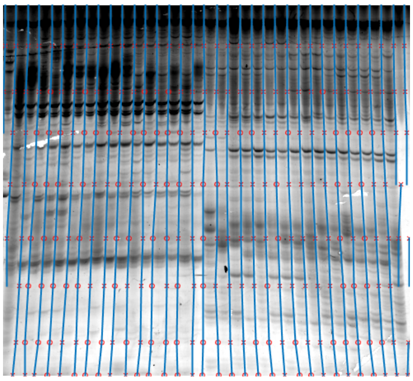
