# Supplementary material for: Automatic DNA Diagnosis for 1D Gel Electrophoresis Images using Bio-image Processing Technique
Source: BMC Genomics. 2015 Dec 9;16(Suppl 12):S15. doi: 10.1186/1471-2164-16-S12-S15 (PMC4682448; doi:10.1186/1471-2164-16-S12-S15)
Supplement: Additional file 2 — Table T1 - Automated lane identification results. Test images shown in Additional File 5: Figure S4 were analyzed using the DNA fingerprinting programs under their default settings for automated lane identification. [file 1471-2164-16-S12-S15-S2.pdf]

Table T1 Automated lane identification results

Image no.	Image dimension	Number of lanes*	PyElph #	GelJ#	GelClust #	GelAnalyzer#	GELect#
1[a]	1884×524	72	6	33	25	58	39
2[a]	1955×524	60	11	37	3	22	37
3[a]	1871×524	72	15	62	38	66	19
4[a]	1911×546	60	6	56	33	53	45
5[a]	1810×718	56	10	36	35	52	46
6[b]	1810×718	34	0	5	0	12	34
7[b]	593×335	32	0	1	0	19	31
8[b]	354×555	15	0	0	0	0	14
9[b]	267×674	15	0	0	0	0	15
10[b]	267×399	17	0	0	0	9	17

Test images shown in Figure S1 were analyzed using the DNA fingerprinting programs under their default settings for automated lane identification.

a Gel images with straight lanes

b Gel images with curved lanes

# The numbers refer to correct lanes assigned automatically by each program.

* The actual number of lanes loaded.
