# Supplementary material for: Automatic DNA Diagnosis for 1D Gel Electrophoresis Images using Bio-image Processing Technique
Source: BMC Genomics. 2015 Dec 9;16(Suppl 12):S15. doi: 10.1186/1471-2164-16-S12-S15 (PMC4682448; doi:10.1186/1471-2164-16-S12-S15)
Supplement: Additional file 3 — Figure S2 - Electrophoresis images used to evaluate lane detection performance. 10 electrophoresis images are used to test the lane detection feature in GELect. [file 1471-2164-16-S12-S15-S3.pdf]

Figure S2 Electrophoresis images used to evaluate lane detection performance.

10 electrophoresis images are used to test the lane detection feature in GELect.

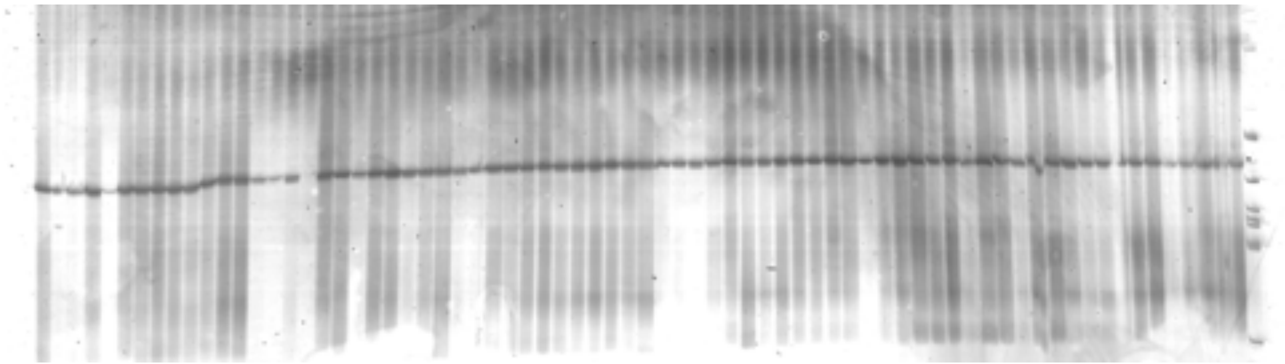

(a) Test image #1

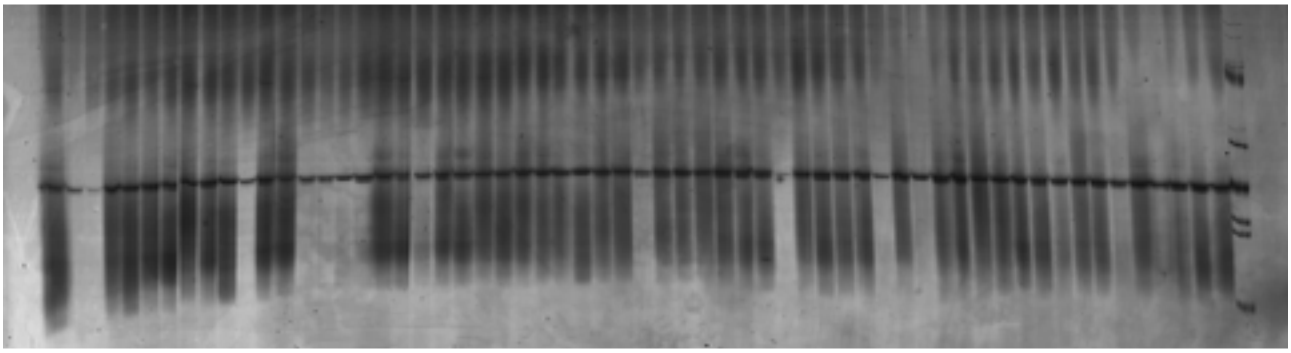

(b) Test image #2

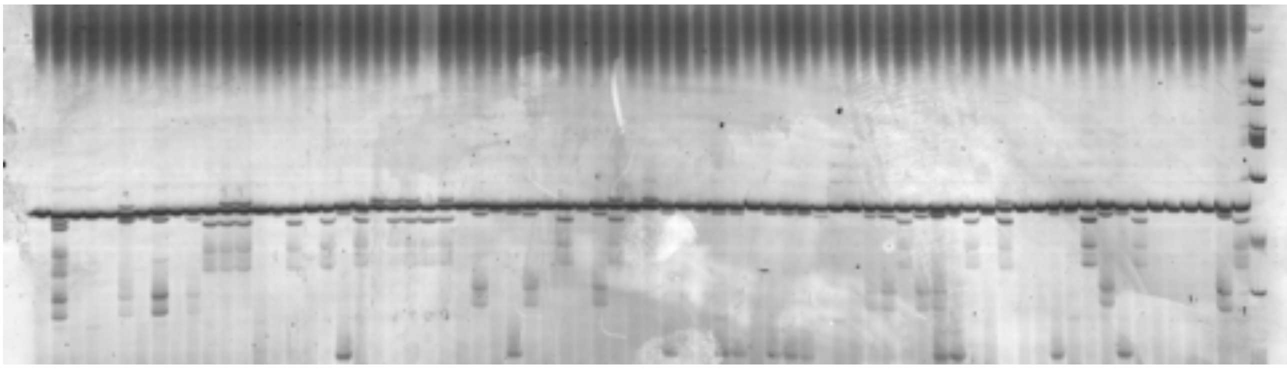

(c) Test image #3

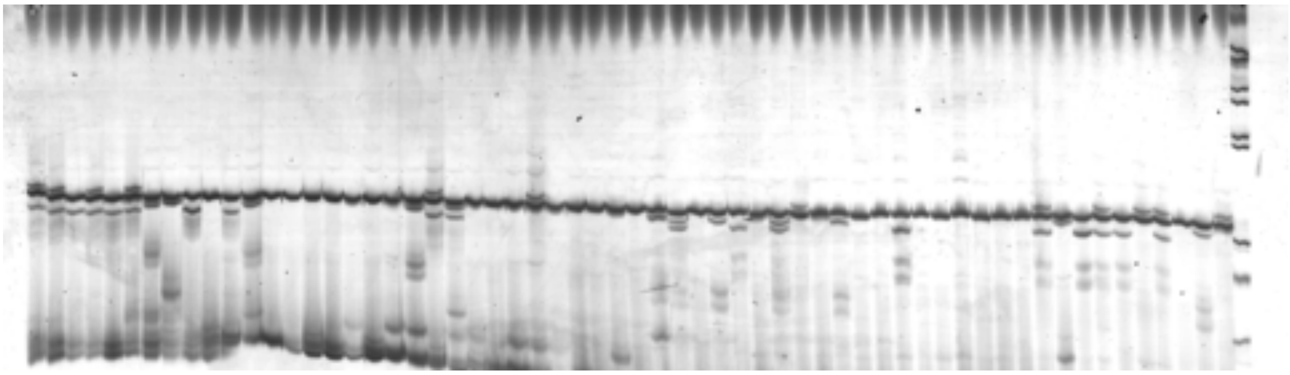

(d) Test image #4

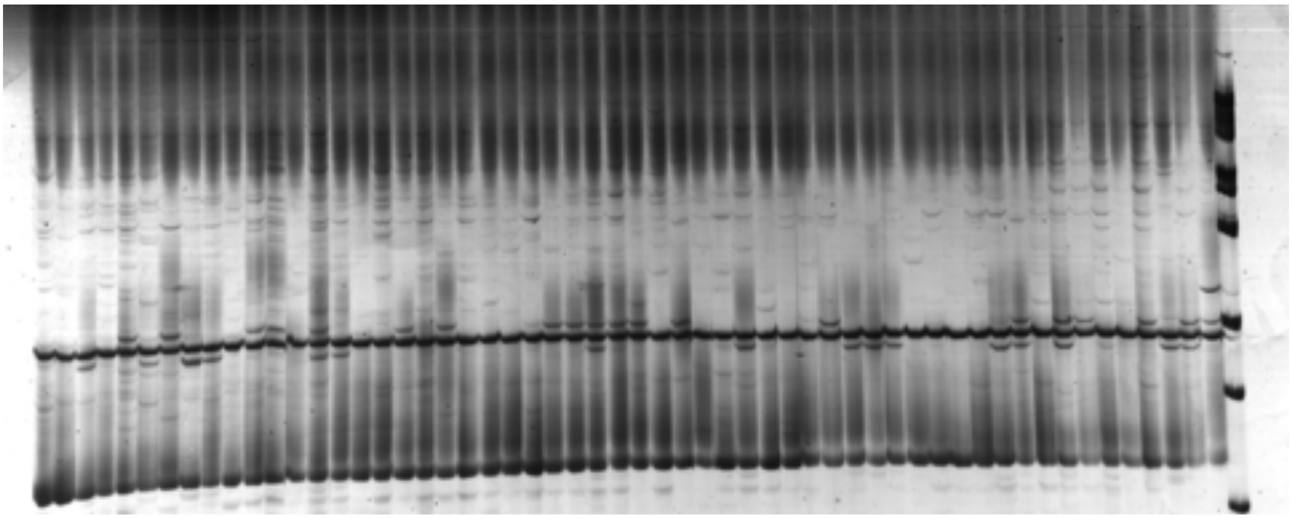

(e) Test image 5

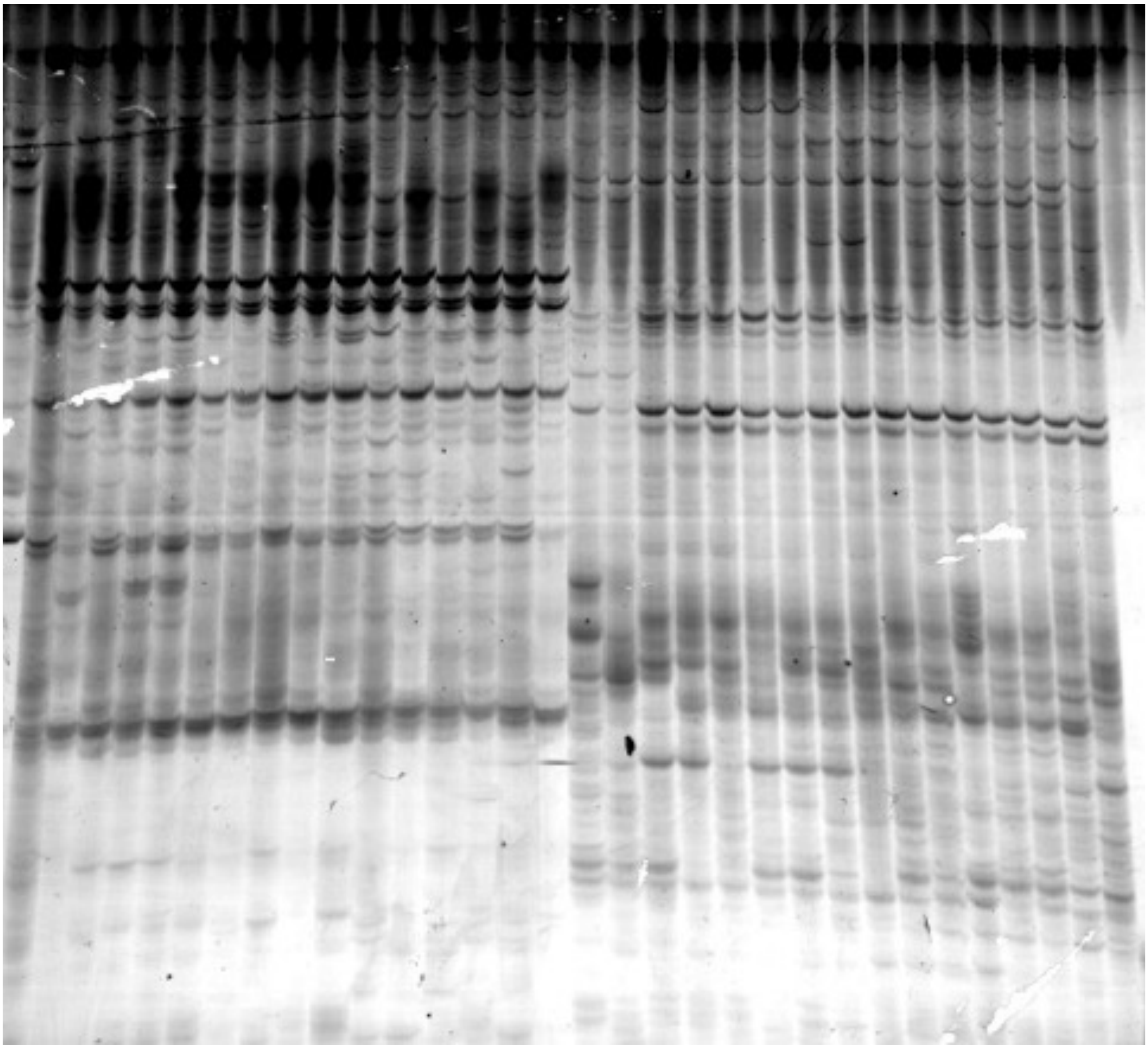

(f) Test image #6

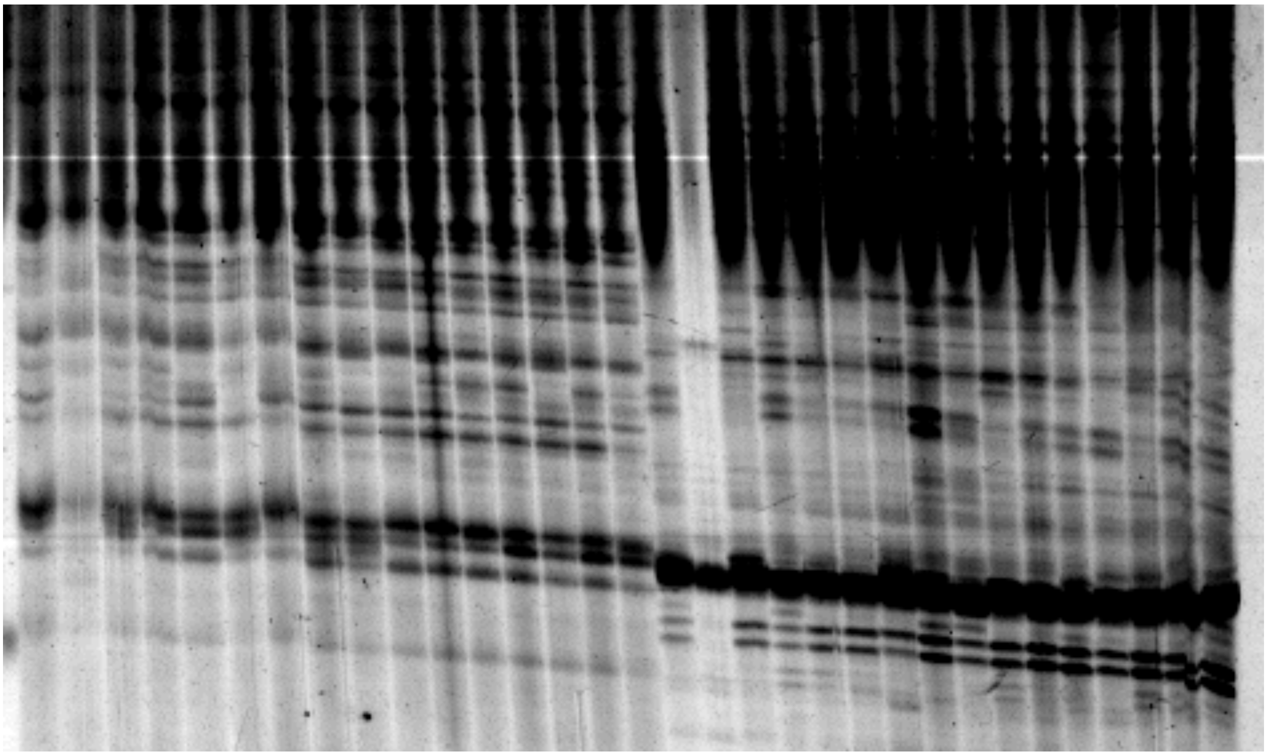

(g) Test image #7

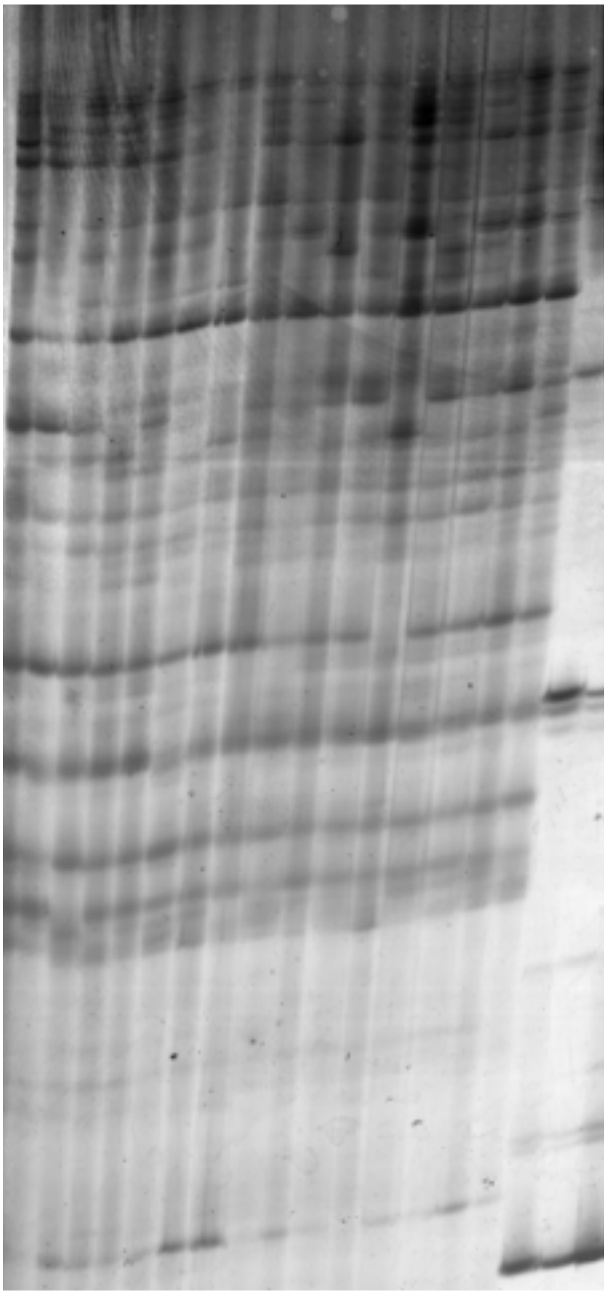

(h) Test image #8

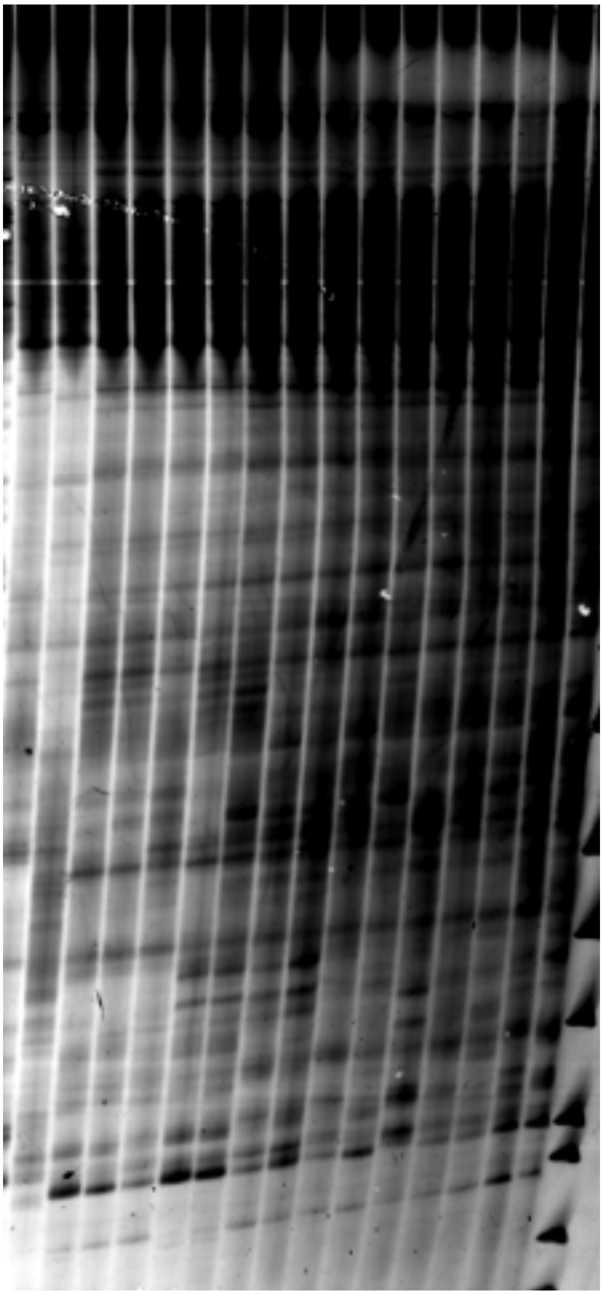

(i) Test image #9

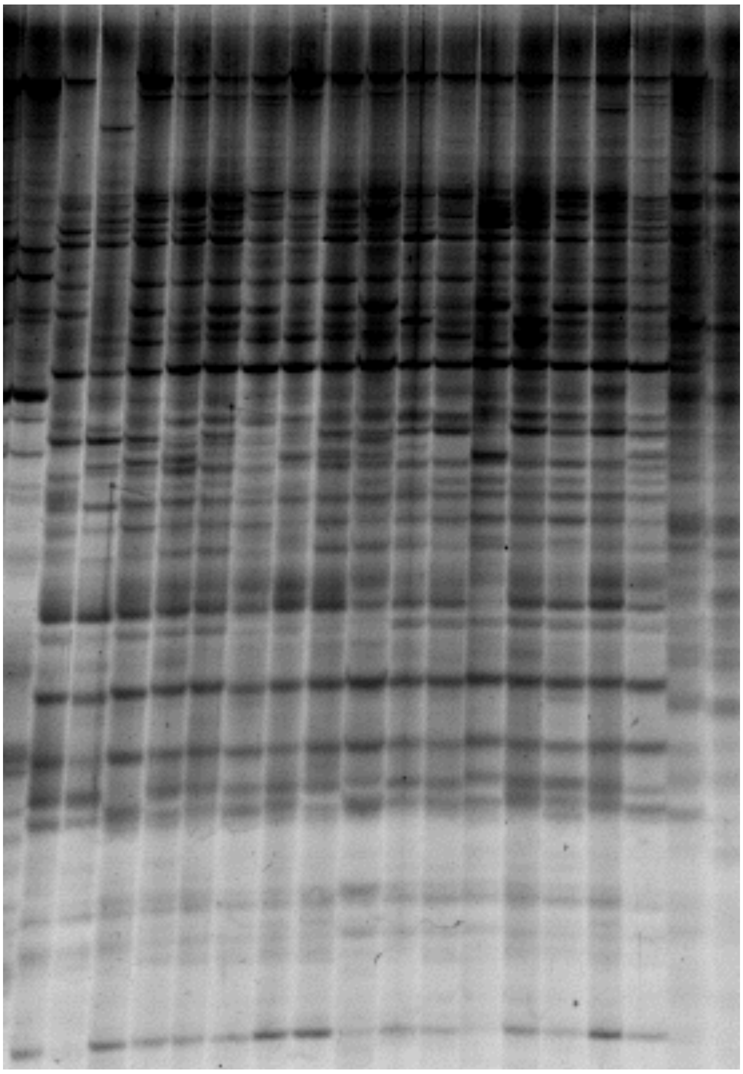

(j) Test image #10
