# Supplementary figures and images for: Automatic DNA Diagnosis for 1D Gel Electrophoresis Images using Bio-image Processing Technique
Source: BMC Genomics. 2015 Dec 9;16(Suppl 12):S15. doi: 10.1186/1471-2164-16-S12-S15 (PMC4682448; doi:10.1186/1471-2164-16-S12-S15)

Figure S3 A diagram shows pixel layout in a typical gel electrophoresis image

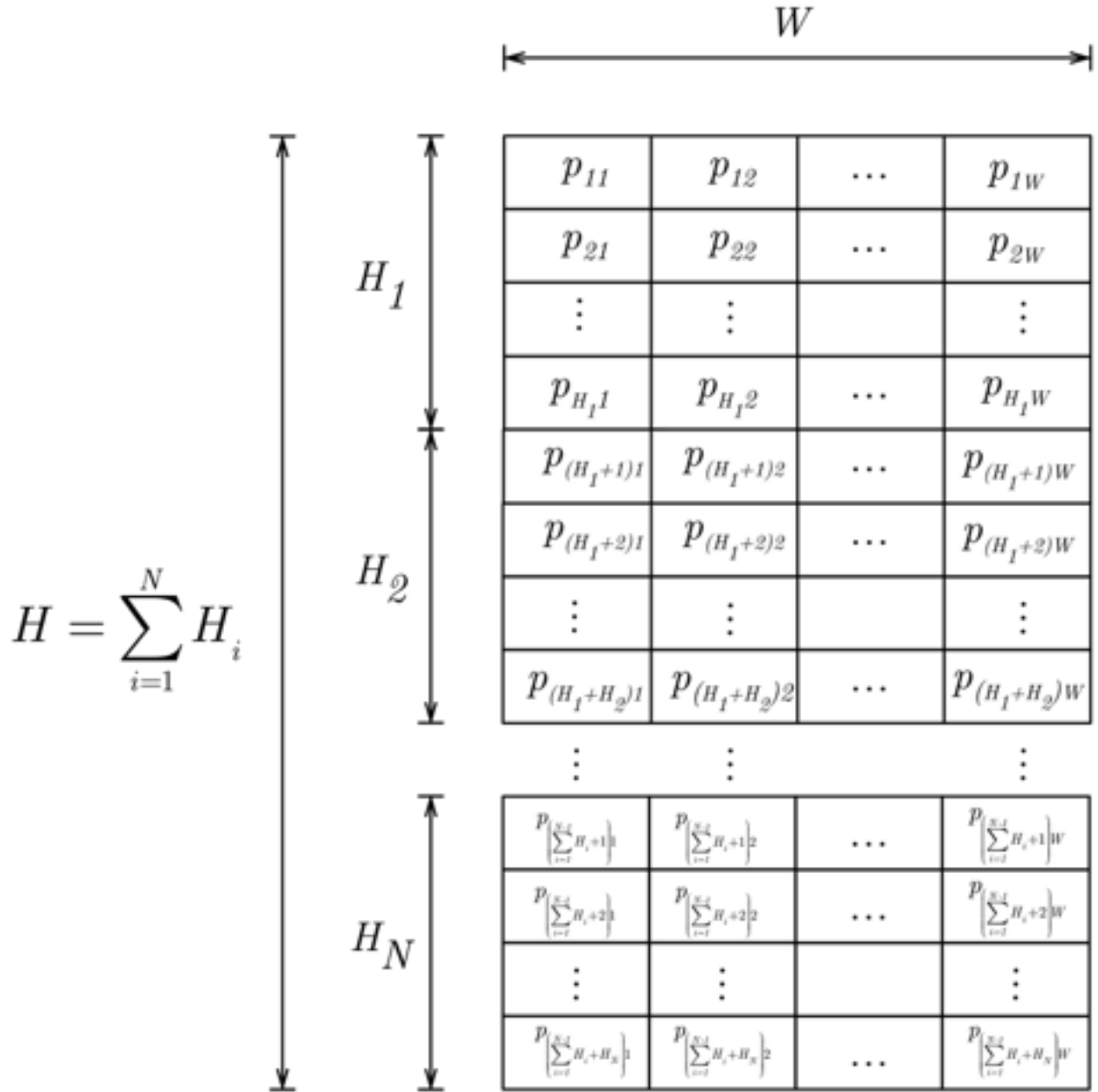

Supplement: Additional file 4 — Figure S3 - A diagram shows pixel layout in a typical gel electrophoresis image. Each box represents a pixel in a typical GE image. The image is separated into N strips with sides Hi and equal width W. [file 1471-2164-16-S12-S15-S4.pdf]
