# Supplementary material for: Automatic DNA Diagnosis for 1D Gel Electrophoresis Images using Bio-image Processing Technique
Source: BMC Genomics. 2015 Dec 9;16(Suppl 12):S15. doi: 10.1186/1471-2164-16-S12-S15 (PMC4682448; doi:10.1186/1471-2164-16-S12-S15)
Supplement: Additional file 5 — Figure S4 - Lane detection results on the 10 experimental GE images. A blue line is used to connect two red dots (obtained from histogram peaks). The two red dots that form the shortest path between adjacent strips (Hi and Hi+1) will be connected using a blue line segment. [file 1471-2164-16-S12-S15-S5.pdf]

Figure S4 Lane detection results on the 10 experimental GE images

A blue line is used to connect two red dots (obtained from histogram peaks). The two red dots that form the shortest path between adjacent strips (Hi and Hi+1) will be connected using a blue line segment.

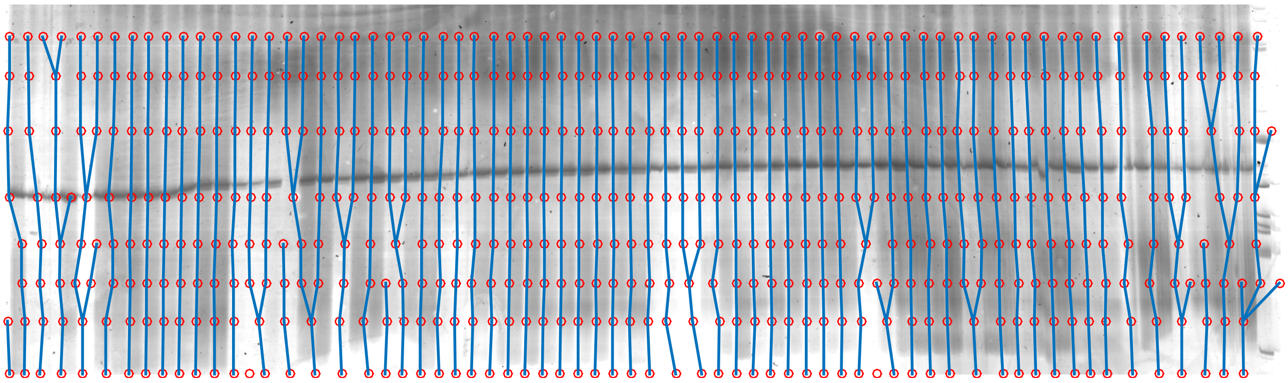

(a) Test image #1 (accuracy 54.16%)

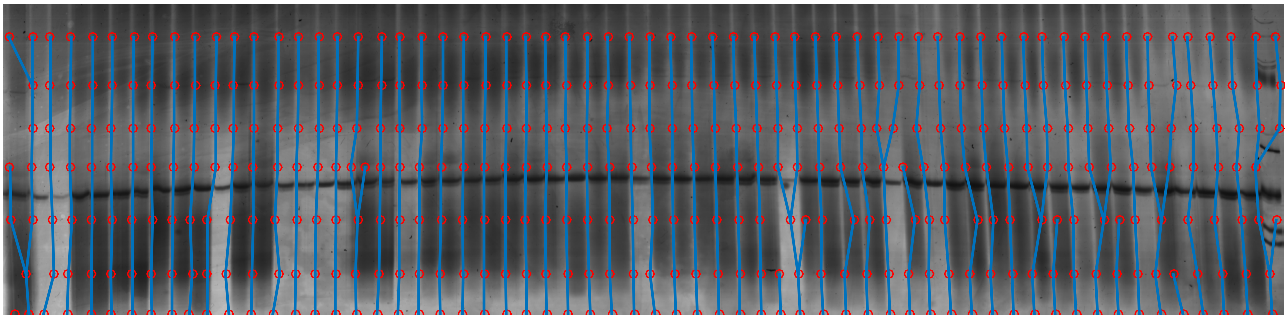

(b) Test image #2 (accuracy 61.67%)

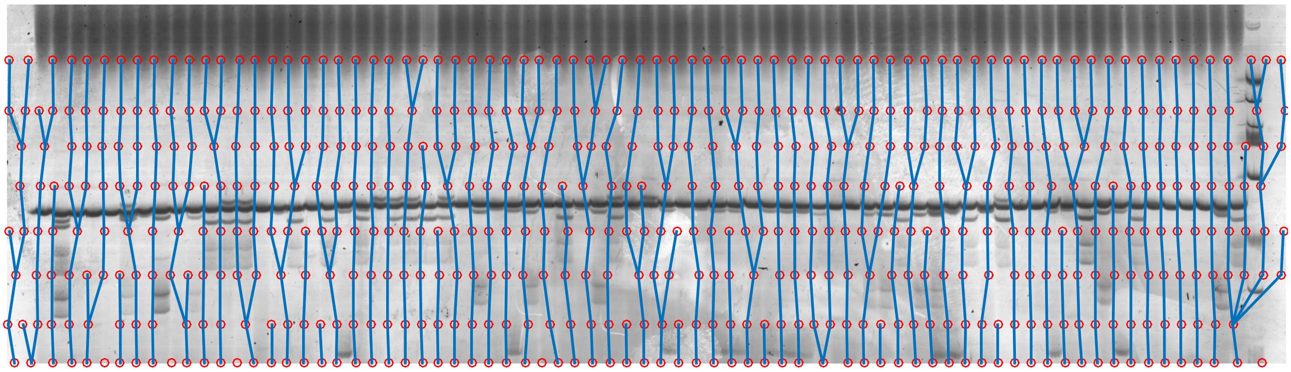

(c) Test image #3 (accuracy 26.83%)

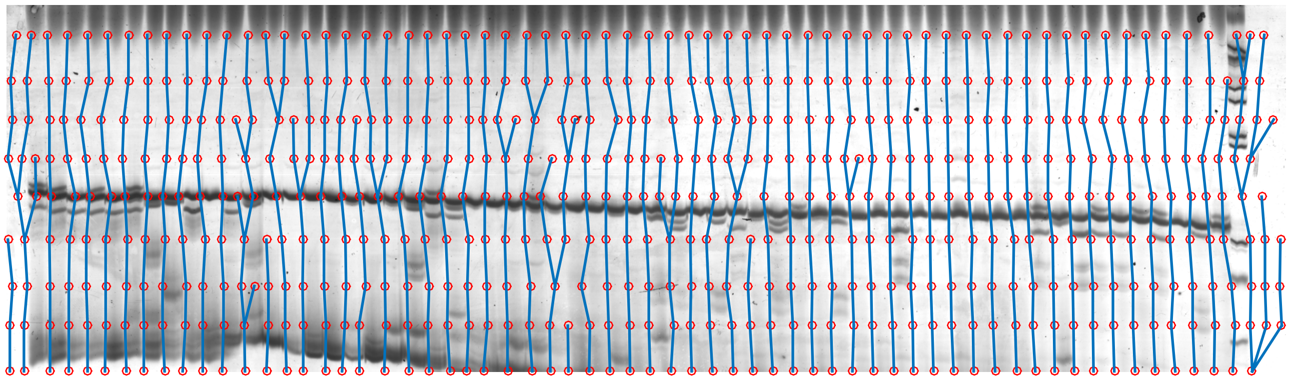

(d) Test image #4 (accuracy 75%)

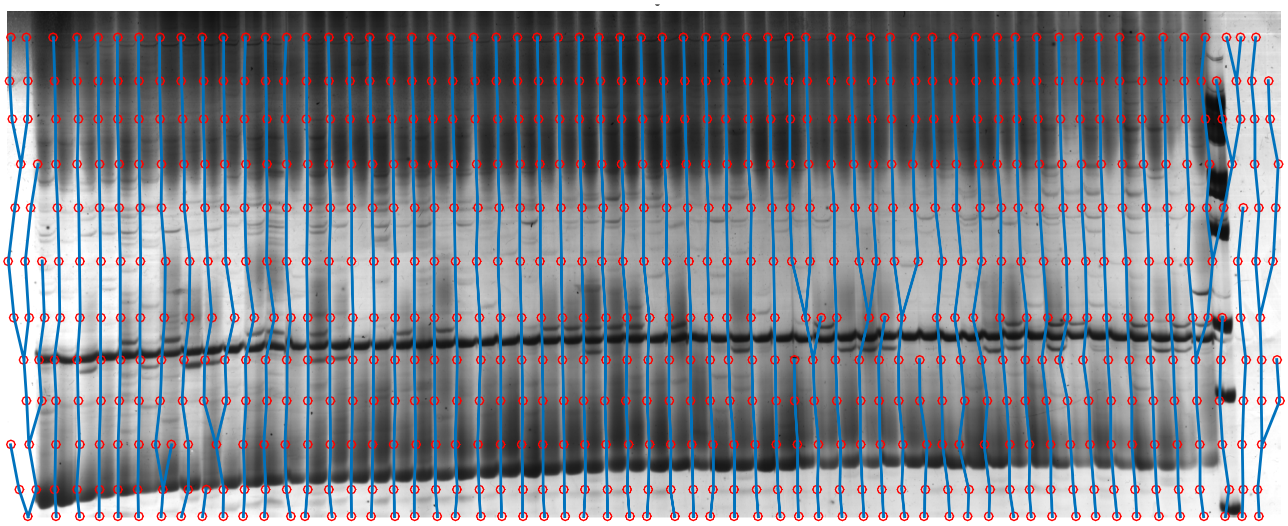

(e) Test image #5 (accuracy 82.14%)

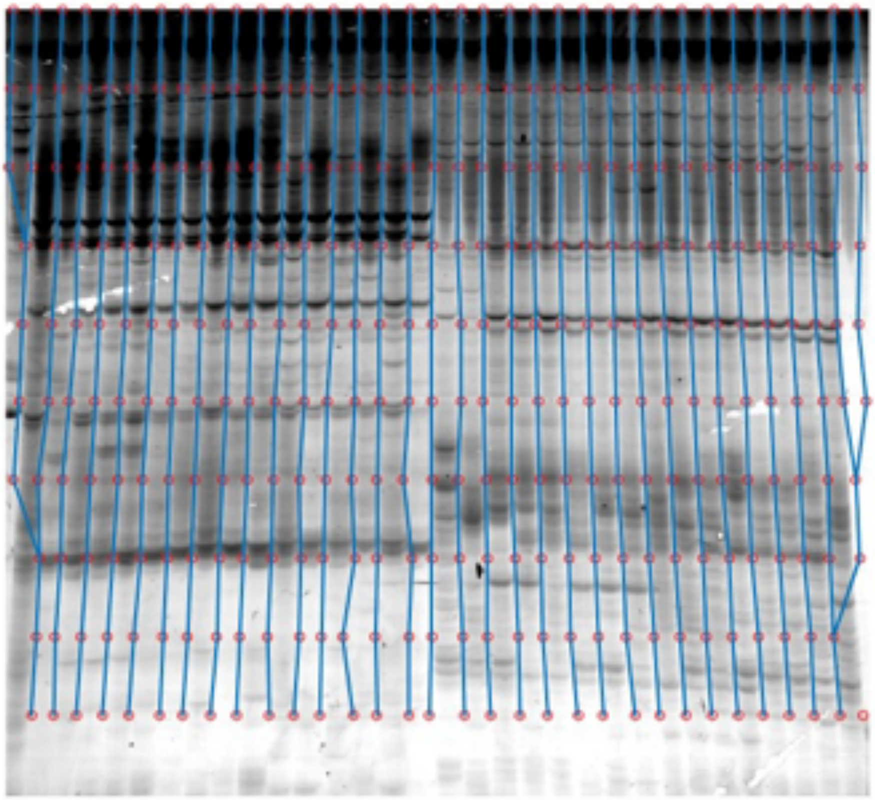

(f) Test image #6 (accuracy 100%)

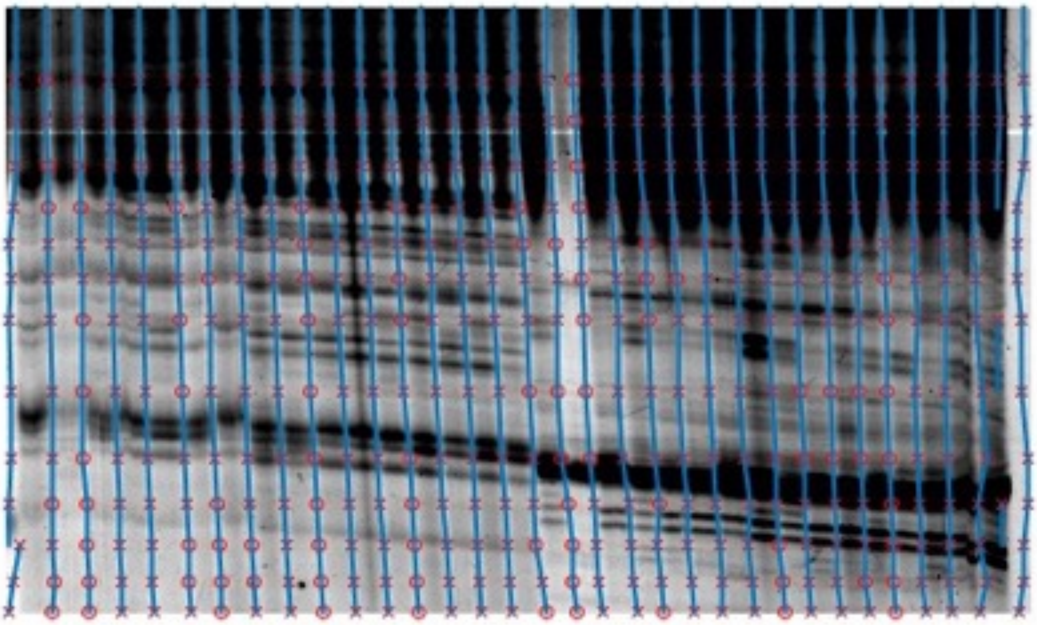

(g) Test image #7 (accuracy 96.87%)

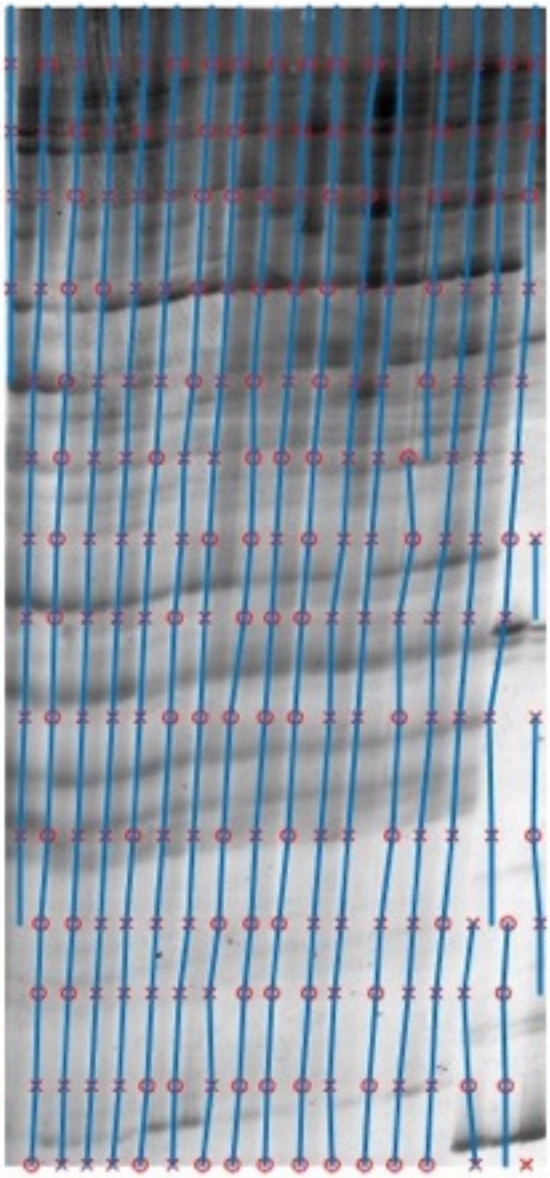

(h) Test image #8 (accuracy 93.33%)

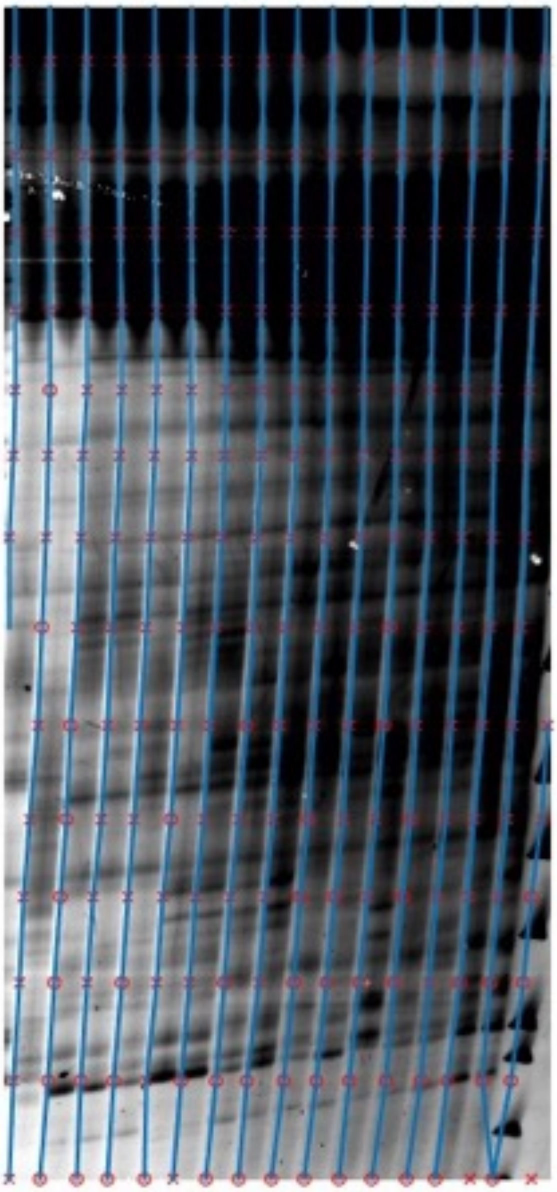

(i) Test image #9 (accuracy 100%)

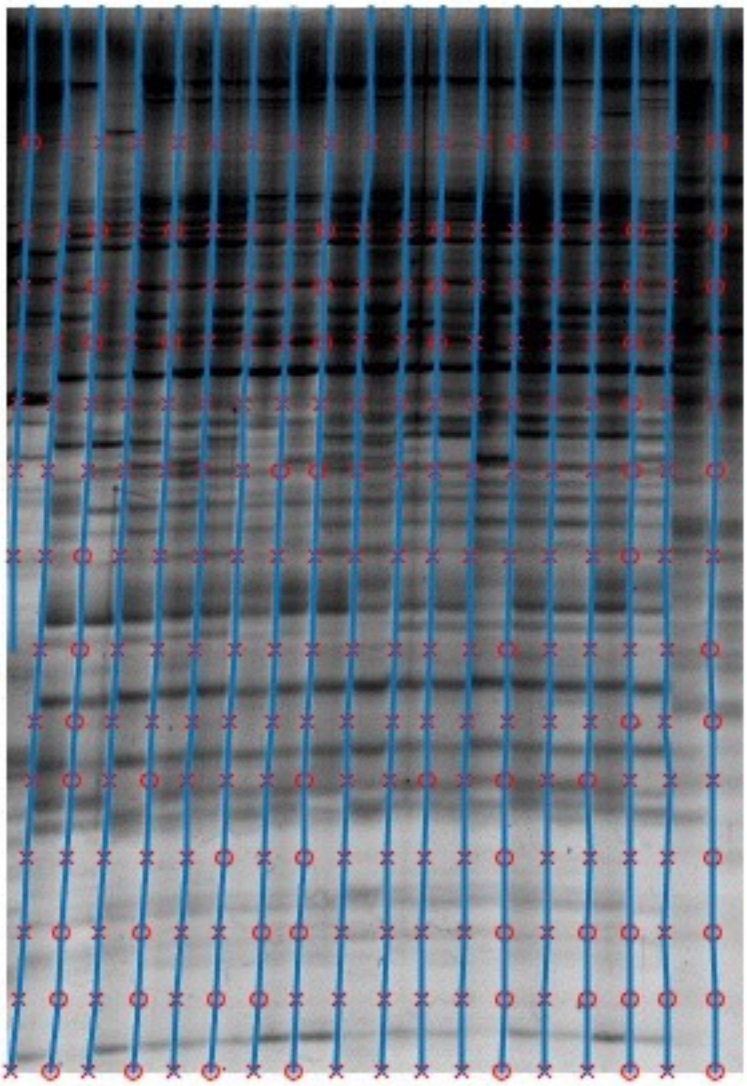

(j) Test image #10 (accuracy 100%)
